# From Six Gene Polymorphisms of the Antioxidant System, Only GPX Pro198Leu and GSTP1 Ile105Val Modulate the Risk of Acute Myeloid Leukemia

**DOI:** 10.1155/2016/2536705

**Published:** 2015-12-28

**Authors:** Claudia Bănescu, Mihaela Iancu, Adrian P. Trifa, Marcela Cândea, Erzsebet Benedek Lazar, Valeriu G. Moldovan, Carmen Duicu, Florin Tripon, Andrei Crauciuc, Minodora Dobreanu

**Affiliations:** ^1^Department of Medical Genetics, University of Medicine and Pharmacy Targu Mures, 38 Gh Marinescu Street, 540139 Targu Mures, Romania; ^2^Department of Medical Informatics and Biostatistics, “Iuliu Hatieganu” University of Medicine and Pharmacy, Cluj-Napoca, 8 Victor Babes Street, 400012 Cluj-Napoca, Romania; ^3^Department of Medical Genetics, “Iuliu Hatieganu” University of Medicine and Pharmacy, Cluj-Napoca, 8 Victor Babes Street, 400012 Cluj-Napoca, Romania; ^4^Hematology Clinic 1, University of Medicine and Pharmacy Targu Mures, 38 Gh Marinescu Street, 540139 Targu Mures, Romania; ^5^Hematology Clinic 2, University of Medicine and Pharmacy Targu Mures, 38 Gh Marinescu Street, 540139 Targu Mures, Romania; ^6^Pediatric Clinic, University of Medicine and Pharmacy Targu Mures, 38 Gh Marinescu Street, 540139 Targu Mures, Romania; ^7^Department of Laboratory Medicine, University of Medicine and Pharmacy Targu Mures, 38 Gh Marinescu Street, 540139 Targu Mures, Romania

## Abstract

Oxidative stress might contribute to the occurrence of cancers, including the hematological ones. Various genetic polymorphisms were shown to increase the quantity of reactive oxygen species, a phenomenon that is able to induce mutations and thus promote cancers. The purpose of the study was to evaluate the association between *CAT* C262T, *GPX1* Pro198Leu, *MnSOD* Ala16Val, *GSTM1, GSTT1,* and *GSTP1* Ile105Val gene polymorphisms and acute myeloid leukemia risk, in a case-control study comprising 102 patients and 303 controls. No association was observed between AML and variant genotypes of *CAT, MnSOD, GSTM1*, and *GSTT1* polymorphisms. Our data revealed a statistically significant difference regarding the frequencies of *GPX1* Pro198Leu and *GSTP1* Ile105Val variant genotypes between AML patients and controls (*p* < 0.001). Our results showed no association in the distribution of any of the *CAT* C262T, *GPX1* Pro198Leu, *GSTM1, GSTT1,* and *GSTP1* polymorphisms regarding age, gender, FAB subtype, cytogenetic risk groups, *FLT3* and *DNMT3* gene mutations, and overall survival. Our data suggests that the presence of variant allele and genotype of *GPX1* Pro198Leu and *GSTP1* Ile105Val gene polymorphisms may modulate the risk of developing AML.

## 1. Introduction

Acute myeloid leukemia (AML) is a complex disease characterized by the accumulation of blasts in the bone marrow and uncontrolled proliferation, in which excess production of oxygen derived radicals compromises the antioxidant defense system thereby leading to oxidative stress [1]. Excessive cellular reactive oxygen species (ROS) or deficiencies in antioxidant defenses are generators of oxidative stress [2], and as previously demonstrated, tumor cells show higher susceptibility to oxidative stress, compared to normal cells.

Oxidative stress has been frequently observed in cancer and was also reported in several hematopoietic malignancies including acute lymphoblastic leukemia [3], acute myeloid leukemia [4], myelodysplastic syndrome (MDS), and chronic myeloid leukemia (CML) [5].

Elevated and persistent ROS levels produce oxidative DNA damage and lead to single-stranded and double-stranded DNA breaks, thus promoting mutagenesis [2].

According to Udensi and Tchounwou, ROS may lead to cancer development by causing gene mutations and/or chromosomal aberrations [6]. Increased production of ROS can lead to acquisition of genomic changes, thereby producing genomic instability. This environment can sustain tumor formation and disease progression [7, 8]. Sallmyr et al. suggested that* FLT3/ITD* mutations (FMS-like tyrosine kinase 3, internal tandem duplications) in acute myeloid leukemia (AML) result in ROS production [7]. This may lead to DNA damage and defective repair mechanisms in myeloid leukemia, besides additional chromosomal aberrations and gene mutations.

The antioxidant defense system includes MnSOD (manganese superoxide dismutase), GPX (glutathione peroxidase), and catalase (CAT) which inactivate ROS [1, 9].

Manganese superoxide dismutase (MnSOD), an antioxidant enzyme, has a critical role in protecting cells against oxidative stress by eliminating superoxide radicals after their conversion to H_2_O_2_ and oxygen [5, 10, 11].

Several* MnSOD* polymorphisms were found to cause an increased superoxide dismutase activity and increased H_2_O_2_ (hydrogen peroxide) quantities that may produce high levels of ROS, which if not subsequently neutralized represent contributing factors to the neoplastic transformation of cells [5, 12].

Glutathione peroxidase (GPX1), an antioxidant defense enzyme, detoxifies hydrogen peroxide into water and oxygen [13]. It has been reported that* GPX1* gene polymorphism (*GPX1* Pro198Leu) is associated with decreased enzyme activity, thereby conferring an increased risk of developing cancer in Caucasians [14].

Catalase (CAT), an important enzyme of the antioxidant system, converts H_2_O_2_ to water and molecular oxygen [15]. The expression of catalase is influenced by gene polymorphisms, which may lead to differences in susceptibility of individuals to oxidative damage caused by ROS [15]. According to an up-to-date meta-analysis performed by Shen et al.,* CAT* C262T polymorphism may be a risk factor for cancer, with cancer type-specific effects [16].

Glutathione S-transferase (GST) enzymes are involved in the metabolism and detoxification of a wide variety of oxidative stress products, xenobiotics, and carcinogens [3].

Glutathione S-transferases (GSTs), phase II metabolizing enzymes, are encoded by* GST* genes and have an important role in cellular defense and therefore in protecting tissues against oxidative damage [5, 17].

*GST* gene polymorphisms are associated with deficiencies in enzyme activity and have been implicated in susceptibility to acute leukemia, but study results are still controversial [4, 18, 19].

To the best of our knowledge, even though the role of oxidative stress and* GSTM1*,* GSTT1*,* GSTP1*,* CAT*, and* GPX1* as well as* MnSOD2* in the pathogenesis of cancer have been previously investigated, no studies on the association of all these six polymorphisms with acute myeloid leukemia have been previously published.

The purpose of the study was to investigate possible associations between glutathione S-transferases (*GSTM1, GSTT1*, and* GSTP1*), superoxide dismutase (*MnSOD* Ala16Val), glutathione peroxidase (*GPX1* Pro198Leu), catalase (*CAT* C262T) gene polymorphisms, and AML susceptibility in a Romanian population.

## 2. Material and Method

### 2.1. Patients and Controls

The research was conducted at the Department of Genetics, University of Medicine and Pharmacy of Targu Mures. The study group consisted of 102 unrelated AML patients (46 males and 56 females; mean age 51.70 ± 16.761 SD, standard deviation, years) and 303 (174 males and 129 females; mean age 46.46 ± 14.501 SD years) unrelated healthy controls with no known malignancies.

The patients were diagnosed with AML according to French-American-British (FAB) subtype and also according to WHO standards [20, 21] at the Hematology Clinics from Targu Mures, Romania, between 2010 and 2013. AML patients were stratified by French-American-British (FAB) subtype as follows: 9 M0, minimally differentiated AML (8.8%); 25 M1, AML without maturation (24.6%); 26 M2, AML with maturation (25.5%); 3 M3, acute promyelocytic leukemia (2.9%); 19 M4, acute myelomonocytic leukemia (18.6%); 15 M5, acute monocytic leukemia (14.7%); 1 M6, erythroleukemia (0.9%); and 4 M7, megakaryoblastic leukemia (3.9%).

Based on WHO 2008 standards and available data, AML cases included in the present study were classified as follows: 14 AML with recurrent cytogenetic anomalies (13.72%), 9 AML dysplasia related (8.82%), 0 myeloid neoplasia therapy related (0%), 0 myeloid sarcoma (0%), 0 myeloid proliferations Down syndrome related (0%), and 79 AML not otherwise specified (77.45%).

Regarding the cytogenetic risk group, favorable group comprised 10 AML patients (9.9%) and intermediate risk group comprised 66 cases (64.7%) while unfavorable (or high risk) cytogenetic group comprised 12 patients (11.7%).

For induction treatment, the standardized protocol consisting of a combination of anthracycline and cytarabine-ara (Ara-C) in the well-known 7 + 3 days' protocol was used. The anthracycline used was daunorubicin or idarubicin. Ara-C was administered as a bolus every 12 hours or continuous infusion over 7 days. All the transretinoic acid (ATRA) was administered in patients with a diagnosis of acute promyelocytic leukemia (APL). In AML, intensive consolidation was used, the standard dose for consolidation being 1.5 g/m^2^ every 12 hours. Reduced intensity conditioning was used in some cases in patients above 60 years of age or in those with poor performance (due to the associated diseases) before the start of chemotherapy.

Both patients and healthy controls were from the central region of Romania. AML cases were followed-up till their death or the beginning of 2015. From the investigated AML patients, 57 cases achieved complete remission (CR) from which 31 relapsed, 23 attained partial remission (PR), and 21 were refractory to treatment.

### 2.2. Genotyping Procedures

Fresh whole blood samples were collected at the time of diagnosis in tubes containing ethylene diamine triacetic acid (EDTA). Genomic DNA was extracted using Quick-gDNA MiniPrep kits (ZymoResearch, USA) and Wizard Genomic DNA Purification kits (Promega, Madison, WI, USA) according to the manufacturers' instructions.

*FLT3* (fms-like tyrosine kinase 3) and* DNMT3A* (DNA methyltransferase) mutations were assessed using PCR-based methods in all AML patients, as previously described [22, 23].

*CAT* C262T*, GPX1* Pro198Leu,* MnSOD* Ala16Val, and* GSTP1* Ile105Val polymorphisms were genotyped by polymerase chain reaction and restriction fragment length polymorphism (PCR-RFLP) methods as previously described with minor modifications to the PCR protocol and digestion step for superoxide dismutase gene which consisted of exposing PCR amplicons obtained by Thermo Scientific FastDigest HaeIII (BsuRI) for 20 minutes [15, 24–26].

Genotyping of the* GSTM1* and* GSTT1* polymorphisms was carried out by multiplex polymerase chain reaction as described by Sharma et al. [27].

### 2.3. Statistical Analysis

Nominal variables were described as absolutes and relative frequencies (%) and the association between them was analyzed by Pearson's Chi-square test or Fisher's Exact Test. The size effect for statistically significant associations was expressed as an odds ratio (OR) with 95% confidence interval associated. Each polymorphism of interest was analyzed univariately as a possible predictor for AML using simple binary logistic regression. The independent effect of a polymorphism was tested using multiple binary logistic regression. Multivariable model was defined considering all exogenous variables whose estimated significance level in univariate logistic regression was *p* < 0.25, the polymorphism's effects being adjusted for possible confounders (gender variable). The best predictive model was chosen comparing nested models based on Akaike information criterion (AIC), Bayesian information criterion (BIC), and Likelihood Ratio Test (LR). All the regression models were additive models that resulted from no significance of interaction terms.

The performance of the final logistic model was evaluated with respect to goodness of fit (Nagelkerke *R* ^2^ coefficient, Brier score), discrimination (predictive and classificatory ability) [28], and calibration aspects [29]. Discrimination was established using *c*-index which is equal to the area under receiver operating characteristic curve (AUC), while Somers' *D* index, discrimination slope, and calibration were analyzed by graphical representation of the concordance between predicted model probabilities and observed proportions of criterion variable. The unreliability index (*U*), quality index (*Q*), maximal error, and mean squared error were used for measuring model miscalibration. In order to assess the reproducibility of the logistic model we performed internal validation by bootstrap resampling method [30]. Using 1000 bootstrap resamples, we calculated unbiased optimism corrected for all estimates of model performance indices.

The Kaplan-Meier estimates were determined for each polymorphism and survival distributions were compared using DeLong test.

The level of statistical significance for all two-sided tests was set at *p* < 0.05.

Statistical analysis was performed with the R software version 3.1.3 (R Foundation for Statistical Computing, Vienna, Austria) using rms libraries.

The study protocol was approved by the Ethics Committees of the University of Medicine and Pharmacy Tirgu Mures and informed consents were signed by the patients.

## 3. Results

*FLT3* mutations were found in 24 AML cases while DNMT3A mutations were identified in 12 patients. The frequency of* CAT* 262T allele in AML cases was 25.9% while in controls it was 26.5% (*p* = 9.269). The frequency of* GPX1* 198Leu allele was significantly higher in AML cases (83.3%) compared to healthy controls (57.2%) (*p* < 0.0001, OR = 3.707, and 95% CI: 2.48–5.541). Variant allele frequencies of the* MnSOD* Ala16Val polymorphism in case and control groups were 47.3% and 54.5%. The frequency of variant* GSTP1* 105Val allele was 33.8% in AML group and 26.9% in control group. There was a significant difference between distributions of* GSTP1* Ile105Val allele frequencies in AML group and control subjects (*p* < 0.0001, OR = 2.357, and 95% CI: 1.649–3.368). In AML patients, the frequencies of* GSTM1* and* GSTT1* null genotypes were 57.8% and 23.5%, respectively. The genotype distributions of* CAT*,* GPX*,* MnSOD*,* GSTP1*,* GSTM1*, and* GSTT1* gene polymorphisms in AML cases and controls are shown in Table 1.

There were no significant differences between variant genotype and AML risk for* CAT* C262T (Chi-square test, *χ* ^2^ = 0.03, df = 2, and *p* = 0.985),* MnSOD2* (Chi-square test, *χ* ^2^ = 4.022, df = 2, and *p* = 0.134),* GSTM1* (Chi-square test, *χ* ^2^ = 0.026, df = 1, and *p* = 0.873), and* GSTT1* (Chi-square test, *χ* ^2^ = 0.339, df = 1, and *p* = 0.560) polymorphisms.

Our data revealed a statistically significant difference regarding the frequencies of* GPX1* Pro198Leu genotypes between AML patients and controls (Chi-square test, *χ* ^2^ = 62.399, df = 2, and *p* < 0.001).

Also, a significant difference in the frequency of* GSTP1* Ile105Val variant genotype was found between AML group and controls (Chi-square test, *χ* ^2^ = 27.606, df = 2, and *p* < 0.001).

The distribution of investigated polymorphisms' combined variant (heterozygous and homozygous) genotype in AML patients stratified by age, gender, FAB subtype, cytogenetic risk group, and* FLT3* and* DNMT3A* mutations criteria is presented in Table 2.

Presence of the variant genotype of* CAT* C262T*, GPX1* Pro198Leu,* MnSOD* Ala16Val, and* GSTP1* Ile105Val, as well as null* GSTM1* and* GSTT1* genotype in AML group was further analyzed in relation to gender and age. There was no significant difference regarding the variant genotype according to gender and age in AML patients (*p* > 0.05 for all these comparisons).

Furthermore, genotype frequencies for all six gene polymorphisms were compared with French-American-British (FAB) subtype and cytogenetic risk group.

No association was found between FAB subtype and some of the investigated gene polymorphisms, namely,* CAT* C262T*, GPX1* Pro198Leu,* GSTP1* Ile105Val,* GSTM1*, and* GSTT1*. A positive association was obtained between* MnSOD* Ala16Val variant heterozygous and homozygous genotype and FAB subtype (*p* = 0.014), precisely acute monocytic leukemia (M5).

In the case of cytogenetic classification according to the risk group, it was not associated with variant genotype of the investigated gene polymorphism (*p* > 0.5 for all comparisons performed).

Furthermore, we analyzed whether there are any associations between* DNMT3A* and* FLT3* gene mutations and investigated gene polymorphisms in AML patients. We observed no significant differences (*p* > 0.05) between AML patients with* DNMT3A* or* FLT3* gene mutations and variant genotype in the case of* CAT* C262T*, GPX1* Pro198Leu,* MnSOD* Ala16Val,* GSTP1* Ile105Val,* GSTM1*, and* GSTT1*.

We also investigated if there are any associations between AML patients' outcome and different parameters such as* DNMT3A*,* FLT3* gene mutations, and variant genotypes of all investigated polymorphisms. Only* FLT3* gene mutations were associated with patient outcome (Pearson Chi-Square test, *p* = 0.011). In addition, from multinomial logistic regression, the presence of* FLT3* mutation was a predictor only for relapse (*p* = 0.005, OR = 4.95, 95% CI: 1.63–15.06, and reference category = CR) not for PR (*p* = 0.451, OR = 1.60, 95% CI: 0.47–5.45, and reference category = CR).

In addition, we analyzed the frequency of combined variant genotypes of* CAT* C262T*, GPX1* Pro198Leu,* MnSOD* Ala16Val,* GSTP1* Ile105Val,* GSTM1*, and* GSTT1* in AML cases and controls.

Taking into account the observed frequencies of different combined genotypes of the investigated polymorphisms in relation to the number of variant genotypes, we tested the hypothesis of leukemia association with the presence of more than two variant genotypes. Our data revealed an association between the presence of leukemia and the presence of more than two variant gene polymorphisms (Chi-square test, *χ* ^2^ = 12.16, df = 4, and *p* = 0.016).

In case of the presence of 3 variant genotypes in our AML patients, the crude OR was 2.45 (95% CI crude OR: 1.23–4.88), in case of 4 variant genotypes the crude OR was 1.79 (95% CI crude OR: 0.89–3.60), in case of 5 variant genotypes the crude OR was 2.72 (95% CI crude OR: 1.20–6.19), and in case of 6 variant genotypes the crude OR was 5.79 (95% CI crude OR: 1.63–20.52).

The level of statistical significance of the estimated regression coefficient associated with each of the independent variables was estimated by univariate logistic regression and is described in Table 3. A statistically significant dependency relation was observed between three variables and leukemia (*p* < 0.05).

Table 4 presents the final logistic model, considered the best predictor of leukemia, consistent with the concerning data. From the set of all considered predictors, only* GSTP1* Ile105Val polymorphism can be considered an independent predictor or an independent risk factor for leukemia (*p* < 0.0001) while* GPX1* Pro198Leu and patient gender had a positive effect on the risk of leukemia but only with a tendency towards statistical significance (*p* = 0.09; *p* = 0.08, resp.).

**Figure 1:**
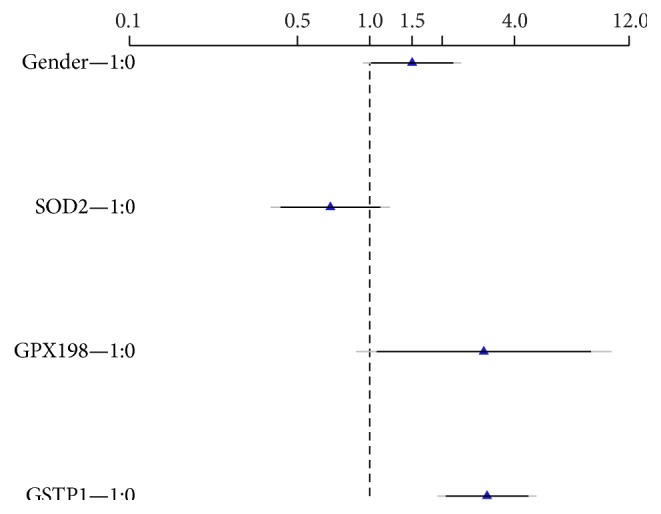
Graphical representation of estimated effects (OR) and associated confidence interval for each factor. Note: black = 90% confidence interval of multivariable adjusted OR; grey = 95% confidence interval of multivariable adjusted OR; 0 = reference category; 1 = variant category.

Figure 1 points out the intensity of the relation between model predictors (gender,* GPX198*, and* GSTP1*) and presence of AML leukemia, the existence of such a relationship being highlighted by a significance level of 0.05 and 0.10.

The model goodness-of-fit indices showed acceptable data fit, the discrimination indices revealed that the model had a good capacity to differentiate between AML subjects and healthy persons, the values close to zero of miscalibration indices showed a well-calibrated nomogram, and the internal validation procedure demonstrated the stability of selection of independent variables (Table 5).

**Figure 2:**
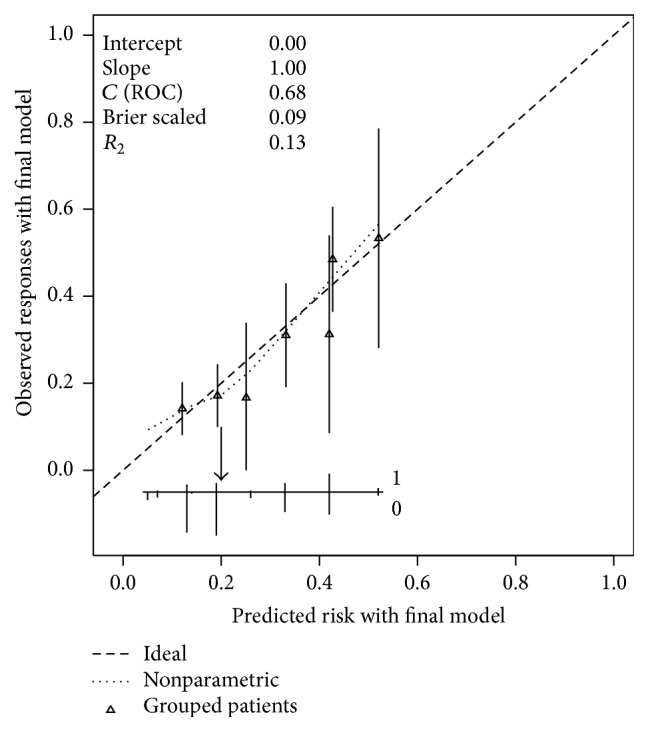
Calibration of the nomogram for AML prediction. The horizontal axis contains the predicted probability of AML; the vertical axis described the observed probability of AML. Perfect prediction corresponds to the dashed oblique line (45°). Bars correspond to CI of estimated grouped proportions and the arrow corresponds to the cut-off of 20% risk of AML.

In Figure 2, the calibration graph suggested a predictive model with an acceptable level of concordance between predicted and observed probabilities.

## 4. Discussion

Certain genetic disorders, environmental carcinogens, physical (ionizing radiation) and chemical exposure, and chemotherapy may lead to acute myeloid leukemia, a heterogeneous disease [18, 31]. Sustained environmental stress may lead to overproduction of ROS and thus significant cell damage and occurrence of somatic mutations. This in turn favors the neoplastic transformation. MnSOD2, GPX, and CAT enzymes are involved in the prevention of DNA damage by ROS [32]. It was reported that* MnSOD2*,* GPX*, and* CAT* gene polymorphisms decrease the enzymatic activity and therefore may increase the risk of cancer by inducing oxidative DNA damage [14, 33].

Also,* GST* gene polymorphisms were associated with a decreased capacity of detoxification for certain mutagens and carcinogens [34].

In the present study, we investigated the oxidative stress enzyme polymorphisms on AML risk in a Romanian population, from the country's central region.

*GST* gene polymorphisms have been extensively studied in AML and* GSTM1* and* GSTT1* null genotypes have been found to increase the risk of AML in both Caucasians and Asians [4, 17, 18, 35, 36].

In the current study, we observed no significant differences between distributions of* GSTM1* and* GSTT1* null genotypes in patients with AML and controls. Similar results were found when we analyzed combined* GSTM1* and* GSTT1* null genotypes. Therefore, we may consider that* GSTT1* and* GSTM1* null genotypes are not associated with the risk of AML in Romanian patients. Our findings are similar to those reported in a recent study performed on chronic myeloid leukemia (CML) in a Romanian population [5].

In contradiction, a recent meta-analysis performed by He et al. showed that* GSTM1* null genotype was associated with the risk of developing AML in East Asians while* GSTT1* null genotype was a risk factor for AML in Caucasians [37]. The same study revealed that the presence of both* GSTM1* and* GSTT1* null genotypes might increase significantly the risk of AML in both Asians and Caucasians [37].

In a study performed on 147 ALL and 143 AML patients by Dunna et al., the homozygous variant genotype of the* GSTP1* Ile105Val polymorphism was associated with the risk of developing acute leukemia and was associated with poor prognosis [19]. In agreement with the previous study, our research indicated that the presence of variant* GSTP1* Ile105Val genotype significantly increases the risk of AML. In contradiction, in a meta-analysis performed by Tang et al.* GSTP1* polymorphism was not associated with acute leukemia risk in Asians [18]. Similar results to those reported by Tang et al. were observed by He et al. [37].

Taking into account that the relationship between all six gene polymorphisms and overall survival has not been previously investigated, we analyzed the effect of predictors (variant genotype) on survival time by using DeLong's test crude *p* values.

Our findings revealed that* GSTP1* Ile105Val variant genotypes and* GSTT1* and* GSTM1* null genotypes did not modify overall survival in AML patients.

Data obtained from our study show that the presence of variant genotypes of* CAT* C262T and* MnSOD* Ala16Val gene polymorphisms is not associated with the risk of AML. Our findings are consistent with a previous study performed on patients with CML from Romania [5]. Similarly, a recent case-study performed on Persian (Caucasians) Muslims living in Shiraz (Iran), a heterogeneous population, and a meta-analysis reported no significant association between* CAT* C262T gene polymorphism and susceptibility to breast cancer [38].

Results from Kaplan-Meyer analysis showed no difference in overall survival between patients with variant and wild-type genotype for* CAT* C262T gene polymorphism or for* GPX1* Pro198Leu gene polymorphism.

Similar results were reported in a research performed on 89 AML patients regarding overall survival between carriers of the variant and wild-type genotypes of* CAT* C262T and* GPX1* Pro198Leu polymorphisms [11].

Regarding* MnSOD* Ala16Val gene polymorphism and overall survival, our results showed that variant genotypes were not associated with significantly shorter survival in AML, compared to the wild-type genotype. Of the studied variables, only* FLT3* mutation has a significant effect on survival time (Cox regression, *p* < 0.001).

In contradiction to our findings, in the study conducted by Koistinen et al. a significant overall survival (*p* = 0.02) was observed for AML patients carrying Val allele of* MnSOD* Ala16Val polymorphism compared to cases with Ala/Ala genotype [11].

Regarding* GPX1* Pro198Leu genotypes, we observed that the presence of variant genotype is associated with a statistically significant risk of AML. Our findings are in contradiction with those previously observed on CML patients from the same region (central part) of Romania [5].

According to Liu et al. in a meta-analysis which included 8.102 patients with breast cancer no statistically significant association was found between* MnSOD* gene polymorphism and risk for breast cancer, excepting the variant allele in premenopausal women [39].

Our study is the first to evaluate the association of all six genes' polymorphisms (*CAT* C262T*, GPX1* Pro198Leu,* MnSOD* Ala16Val,* GSTT1*,* GSTM1*, and* GSTP1* Ile105Val) with AML among de novo patients.

Univariate logistic regression revealed that combined variant (heterozygous + homozygous) genotype of* CAT, GPX1, MnSOD,* and* GSTP1* gene polymorphisms is associated with an increased risk of developing AML (*p* = 0.003, OR = 12.68, and 95% CI: 2.36–68.01).

A positive association was observed between combined variant (heterozygous + homozygous) genotype of* CAT, GPX1, MnSOD,* and* GSTP1* and null genotype for* GSTM1* and AML risk (*p* = 0.017, OR = 7.34, and 95% CI: 1.43–37.67). We have found a statistically significant correlation between variant genotypes for* GPX1* Pro198Leu,* MnSOD* Ala16Val,* GSTP1* Ile105Val, and* GSTM1* null genotype and AML in our study (*p* = 0.004, OR = 10.33, and 95% CI: 2.12–50.26). Based on our data, we may suggest that the presence of one of the three possible combined genotypes may represent a risk factor for AML.

We could not find a statistically significant association in the distribution of any of the six gene polymorphisms regarding gender, cytogenetic risk group, and* FLT3* and* DNMT3A* gene mutations.

To our knowledge, the relationship between the investigated gene polymorphisms and* FLT3* and* DNMT3A* gene mutations and AML FAB subtype has not been previously investigated.

In addition, this study is the first on the distribution of* CAT* C262T*, GPX1* Pro198Leu,* MnSOD* Ala16Val,* GSTT1*,* GSTM1*, and* GSTP1* Ile105Val polymorphisms in Romanian AML patients.

Our study has some limitations, such as the relatively small group size, the lack of investigation of* RUNX1* gene mutation (and other mutations), and the lack of antioxidant enzyme activity determination.

In conclusion, our present study reveals that the presence of variant allele and genotype of* GPX1* Pro198Leu and* GSTP1* Ile105Val gene polymorphisms may increase the risk of developing AML. As the number of patients is small, additional studies performed on larger cohorts are required to establish the relationship between these polymorphisms and AML risk.

## Figures and Tables

**Table 1:** Distribution of *CAT*, *GPX*, *MnSOD*, *GSTP1*, *GSTM1*, and *GSTT1* genotypes among AML patients and controls.

Polymorphism	AML patients *n* (%)	Controls *n* (%)	*p* value, OR (95% CI) AML versus controls
*CAT C262T *			
CC	55 (53.9)	161 (53.1)	Reference
CT	41 (40.2)	123 (40.6)	0.985, 0.98 (0.61–1.56)
TT	6 (5.9)	19 (6.3)	0.985, 0.92 (0.35–2.43)
CT + TT	47 (46.1)	142 (46.9)	0.89, 0.969 (0.618–1.519)
*GPX1 Pro198Leu*			
Pro/Pro	3 (2.9)	34 (11.2)	Reference
Pro/Leu	28 (27.5)	190 (62.7)	0.588, 1.670 (0.480–5.80)
Leu/Leu	71 (69.6)	79 (26.1)	<0.0001, 10.186 (2.997–34.622)
Pro/Leu + Leu/Leu	99 (97.1)	269 (88.8)	0.012, 4.171 (1.252–13.886)
*MnSOD Ala16Val*			
Ala/Ala	24 (23.5)	54 (17.8)	Reference
Ala/Val	60 (58.8)	168 (55.4)	0.464, 0.803 (0.457–1.413)
Val/Val	18 (17.6)	81 (26.7)	0.074, 0.50 (0.248–1.009)
Ala/Val + Val/Val	78 (76.5)	249 (82.2)	0.206, 0.705 (0.409–1.214)
*GSTP1 Ile105Val*			
Ile/Ile	39 (38.2)	205 (67.7)	Reference
Ile/Val	57 (55.9)	88 (29.0)	<0.001, 3.405 (2.111–5.491)
Val/Val	6 (5.9)	10 (3.3)	0.0393, 3.154 (1.083–9.183)
Ile/Val + Val/Val	63 (61.8)	98 (32.3)	<0.0001, 3.379 (2.120–5.386)
*GSTM1 *			
Present	43 (42.2)	125 (41.3)	Reference
Null	59 (57.8)	178 (58.7)	0.873, 0.964 (0.612–1.518)
*GSTT1*			
Present	78 (76.5)	240 (79.2)	Reference
Null	24 (23.5)	63 (20.8)	0.56, 1.172 (0.686–2.002)

**Table 2:** AML patient characteristics according to the *CAT *C262T*, GPX1 *Pro198Leu, *MnSOD *Ala16Val, *GSTP1* Ile105Val, *GSTM1*,and *GSTT1 *genotypes.

	*CAT *C262T			*GPX1 *Pro198Leu			*MnSOD *Ala16Val			*GSTP1* Ile105Val			*GSTM1*			*GSTT1*		
	CC	CT + TT	*p* value	Pro/Pro	Pro/Leu + Leu/Leu	*p* value	Ala/Ala	Ala/Val + Val/Val	*p* value	Ile/Ile	Ile/Val + Val/Val	*p* value	Present	null	*p* value	Present	null	*p* value
Gender																		
Female	22	24	Ref	0	46	Ref	13	33	Ref	23	23	Ref	18	28	Ref	36	10	Ref
Male	33	23	0.264	3	53	0.055	11	45	0.308	16	40	0.026	25	31	0.574	42	14	0.699
Age																		
<60 years	37	31	Ref	1	67	Ref	17	51	Ref	24	44	Ref	24	44	Ref	53	15	Ref
>60 years	18	16	0.888	2	32	0.257	7	27	0.805	15	19	0.387	19	15	0.057	25	9	0.805
Age (median, range)																		
<50 years	27	20	Ref	1	46	Ref	13	34	Ref	16	31	Ref	17	30	Ref	35	12	Ref
>50 years	28	27	0.554	2	53	1.00	11	44	0.483	23	32	0.540	26	29	0.316	43	12	0.815
FAB classification			0.698			0.565			0.014			0.333			0.149			0.461
M0	2	4		0	6		2	4		2	4		1	5		6	0	
M1	15	10		2	23		2	23		10	15		10	15		18	7	
M2	16	10		0	26		10	16		5	21		12	14		19	7	
M3	2	1		0	3		1	2		1	2		1	2		3	0	
M4	11	8		0	19		6	13		11	8		5	14		16	3	
M5	6	9		1	14		0	15		6	9		11	4		10	5	
M6	1	3		0	4		1	3		2	2		1	3		3	1	
M7	2	2		0	4		2	2		2	2		2	2		3	1	
Cytogenetic risk group			0.058			0.549			0.067			0.925			0.630			0.194
Favorable	6	6	Ref	0	12	Ref	4	8	Ref	4	8	Ref	6	6	Ref	11	1	Ref
Intermediate	38	26	0.751	2	62	1.00	11	53	0.238	25	39	1.00	27	37	0.753	48	16	0.279
Unfavorable	2	8	0.204	0	10	—	5	5	0.665	4	6	1.00	3	7	0.730	6	4	0.136
ND	9	7	1.00	1	15	1.00	4	12	0.691	6	10	1.00	7	9	1.00	13	3	0.613
FLT3 mutations																		
FLT3−	40	38	Ref	3	75	Ref	17	61	Ref	31	47	Ref	36	42	Ref	57	21	Ref
FLT3+	15	9	0.93	0	24	1.00	7	17	0.457	8	16	0.572	7	17	0.141	21	3	0.145
DNMT3A mutations																		
DNMT3A−	48	42	Ref	2	88	Ref	21	69	Ref	31	59	Ref	39	51	Ref	69	21	Ref
DNMT3A+	7	5	0.744	1	11	0.316	3	9	1.00	8	4	0.054	4	8	0.510	9	3	1.00

ND: not determined; Ref: reference.

**Table 3:** The effect of predictors on outcome variable (results from univariate logistic regression).

Variables	Statistics *Z*	*p* ^*∗*^	Crude OR	95% CI for crude OR
Gender	2,152	0,031	1,64	1,05–2,58
CAT262	−0,138	0,89	0,97	0,62–1,52
MnSOD2	−1,260	0,208	0,70	0,41–1.21
GPX198	2,328	0,02	4,17	1,25–13,88
GSTM1	−0,160	0,873	0,96	0,61–1,52
GSTT1	0,582	0,561	1,17	0,69–2,00
GSTP1	5,118	<0,001	3,38	2,12–5,39

^*∗*^Wald's test crude *p* values; reference categories: gender = women; CAT262 = normal; SOD2 = normal; GPX198 = normal; GSTM1 = present; GSTT1 = present; GSTP1 = present.

**Table 4:** The final multivariate logistic model.

Variables	*b* ^*∗*^	SE	*p* ^+^	Adjusted OR	95% CI for adjusted OR
Gender	0,41	0,24	0,092	1,5	0,94–2,41
SOD2	−0,38	0,29	0,196	0,69	0,39–1,22
GPX198	1,09	0,63	0,081	2,98	0,88–10,15
GSTP1	1,12	0,24	<0,0001	3,08	1,92–4,94
Constant	−2,54	0,67	0,0001	0,08	0,02–0,26

^*∗*^Estimated unstandardized regression coefficients; SE = standard error; ^+^Wald's test adjusted *p* value.

**Table 5:** Assessment of final model fit.

Performance measure	Final predictive model	Internal validation^*∗*^
Global measure of goodness of fit		
Brier	0,17	0,18
*R* ^2^ (Nagelkerke)	0,13	0,10
Discrimination		
AUC = *C* stat (95% CI)	0,68 (0,63–0,75)	0,67
Somers' *D* index	0,36	0,34
Discrimination slope	1,00	0,90
Calibration		
Hosmer-Lemeshow goodness-of-fit test (*χ* ^2^, *p* value)	6,29 (0,39)	
Unreliability index *U*	−0,005	0,004
Quality index *Q*	0,09	0,06
Maximal error	<0,001	0,04
Mean squared error	0,00061	0,00057

^*∗*^Evaluated by bootstrapping method (number of resampling, *B* = 1000); the optimism corrected indices values.
